# Extraoral Root-End Resection May Promote Pulpal Revascularization in Autotransplanted Mature Teeth—A Retrospective Study

**DOI:** 10.3390/jcm11237199

**Published:** 2022-12-03

**Authors:** Petra Rugani, Barbara Kirnbauer, Irene Mischak, Kurt Ebeleseder, Norbert Jakse

**Affiliations:** Department of Dental Medicine and Oral Health, Division of Oral Surgery and Orthodontics, Medical University of Graz, Billrothgasse 4, 8010 Graz, Austria

**Keywords:** autotransplantation, revascularization, pulpal healing, root-end resection, apicoectomy, controlled clinical trial

## Abstract

Tooth germ autotransplantation of open apices of the teeth exhibits high pulpal healing rates, whereas that of mature permanent teeth normally causes irreversible pulpal necrosis. Extraoral root-end resection (EORER) during transplantation may promote pulpal revascularization (PRV) in transplanted mature teeth and reduce endodontic treatment requirement. This study compared the primary outcomes of survival rates, PRV, and root resorption and determined relevant confounders in autotransplanted mature and immature teeth. The medical charts of consecutive patients who underwent tooth autotransplantation between January 2017 and March 2021 were evaluated. Teeth with a documented follow-up of at least 1 year were included. During the study period, 59 teeth were transplanted in 44 patients. Overall, 2 teeth were excluded owing to missing data; 57 teeth were analyzed, including 25 mature teeth additionally treated with EORER. After a mean follow-up of 21.2 ± 16.1 months, no significant differences in primary outcomes were detected. Fifty-five teeth remained in situ (96.5%), and radiological signs of root resorption were detected in 9/57 teeth (15.8%). PRV was positive in 54/57 teeth (94.7%). Surgical duration and PRV failure were significantly associated with high incidences of root resorption. Mature teeth autotransplantation with EORER yielded similar results to immature teeth autotransplantation and is a feasible treatment option. Long surgery and failed revascularization increased root resorption rates. More factors should be evaluated in larger trials with longer observation periods.

## 1. Introduction

Autotransplantation (AT) refers to the transplantation of a tooth within a patient’s mouth based on tooth extraction and repositioning following surgical bone preparation. The AT of immature teeth with open apices is an established procedure, particularly for post-traumatic treatment and cases of agenesis among older children and adolescents. AT is advantageous primarily because it can prevent bone atrophy and growth retardation at the recipient site owing to the restoration of the physiological stimulation of the jawbone by the transplanted tooth and its periodontium [1]. Hence, it is a preferred treatment option for the growing jaw. In adults, in addition to fixed bridgeworks, resin-bonded restorations, removable partial dentures, and implant therapy constitute the common the treatment of choice. Nevertheless, AT might be a biological alternative if patients prefer such a method or if healthy teeth need to be removed, such as in in cases of orthodontic therapy. Further, it may postpone the need for implant therapy and prevent associated risks in a well-preserved ridge in later life [2,3,4].

The AT of mature teeth can achieve survival rates (≥90%) similar to those of the AT of immature teeth [5,6,7]. To address the crucial difference between mature and immature transplanted teeth, one must distinguish between periodontal and endodontic healing. Periodontal healing following AT is an outcome of proper and adequate surgical technique. The gentle manipulation of the donor tooth [8] and minimal extraoral time [1] are essential for preserving the vital periodontal ligament cells. However, the surgeon’s influence on endodontic healing, including pulp revascularization (PRV) of the transplant, is limited. Endodontic healing may depend on several factors, including the root canal anatomy and apical constriction width [9,10].

The possibility of PRV in immature teeth is good and is further facilitated by a large apical foramen diameter and a high number of apical stem cells [11,12]. A diameter of 0.2–0.4 mm, as found in the apical constrictions of mature premolars, reduces the PRV rate to almost zero [13]. The unlikeliness of endodontic healing is the major shortcoming in the transplantation of mature teeth. Endodontic failure often engenders rapid infection-related external root resorption. Thus, root canal treatment should be performed within 7–14 days in mature transplanted teeth [6,14].

In 1981, Skoglund and Tronstad [15] hypothesized that extraoral root-end resection (EORER) facilitates replanted mature teeth revascularization due to the tapering of the root canal, which removes the narrowest parts of the root canal and increases apical access for cell and vessel invasion. However, the study using a dog model failed to prove this theory.

In 2018, Jakse et al. [16] reported a clinical case of mature premolar transplantation combined with EORER that induced pulp revascularization. Another case [17] employing similar techniques was reported, followed by a retrospective study including 10 transplanted and root-end resected mature teeth [18]. Thus, successful revascularization can obviate the need for root canal treatment and thereby eliminates the main disadvantage in transplantation of mature teeth. [16,18].

Herein, we aimed to compare the outcomes following the AT of immature teeth and mature teeth (combined with EORER). Primary outcomes included tooth survival, root resorption, and PRV. Factors that may influence outcomes, including general health, age, sex, donor type, recipient site, duration of surgery, period of splinting, and stage of root development, were evaluated.

## 2. Materials and Methods

All patients who provided informed consent for the therapeutic options, including for the AT of immature teeth or mature teeth, between January 2017 and March 2021, were included in the study.

The inclusion criteria were patients with sufficient data of clinical and radiological follow-up for at least 1 year and those who underwent radiological preoperative analysis and planning with cross-sectional imaging. Radiological planning included the fabrication of a 3D replica of the transplanted tooth based on segmented cone-beam computed tomography (CBCT) data. Surgeries were performed under local anesthesia by four different senior oral surgeons with extensive experience. During surgery, the 3D replica was used to validate sufficient recipient site preparation using an implant drill kit. Following cautious tooth extraction, immature teeth were immediately transferred to the prepared socket. The root tip of the mature teeth was extra-orally resected using a diamond disk under continuous liquid cooling. Subsequently, the transplant was immediately transferred into the previously prepared recipient site. In cases of tooth impaction, the recipient site was prepared following tooth removal to prevent transplant damage. The teeth were stored in a nutrition media (DENTOSAFE^®^; Medice, Iserlohn, Germany). Following pressure-free insertion, the transplant was fixated in the socket via suturing, and inactive splinting to the neighboring teeth was performed using a trauma splint (TTS Trauma Splint, Medartis, Basel, Switzerland) or flexible wire (GAC Wildcat Wire 0175”, Ortho-Care, Shipley, UK). Post-operative splinting of the transplant was performed for at least 4 weeks.

Patients received perioperative antibiotic prophylaxis for 4 days and antiphlogistic therapy for 2–5 days. Sutures were removed after 1 week. A close follow-up was performed. Patients were recalled every 3 months during the first year and annually thereafter.

Outcome data were collected retrospectively from patient records and dental radiographs, including those of survival, PRV, root resorption, general health (American Society of Anaesthesiologists Physical Status System [ASA]), age, sex, donor type, recipient site, duration of surgery, period of splinting, and stage of root development. CBCT was used to measure the diameter of the apical openings of the transplanted teeth with an accuracy of 200 µm. For EORER, the root canal diameter was assessed at the resection level. The following clinical parameters were used to assess the endodontic outcome: presence/absence of local swelling or sinus tract, sensitivity to percussion, and pulpal sensitivity to an electric pulp tester or carbon dioxide snow. Intraoral radiographs and orthopantomograms were analyzed to determine pulp canal obliteration (PCO, a sign of revascularization), external inflammatory root resorption, and apical radiolucency (a sign of infected pulpal necrosis). If available, data from perfusion magnetic resonance imaging (MRI), performed after splint removal to assess PRV in several cases, were acquired.

Statistical analyses were performed using SPSS software (IBM SPSS statistics 26.0, IBM Corporation, Armonk, New York, NY, USA) with a 5% significance level. Chi-square tests were used for quantitative analyses. Fisher’s exact tests and Chi-square tests were used to analyze categorical data. Independent Student’s t-tests were applied to continuous variables. Logrank test was performed to compare the time until the onset of root resorption in mature and immature teeth. The local ethics committee approved the study protocol (Review board number 33-578 ex 20/21).

## 3. Results

**Figure 1:**
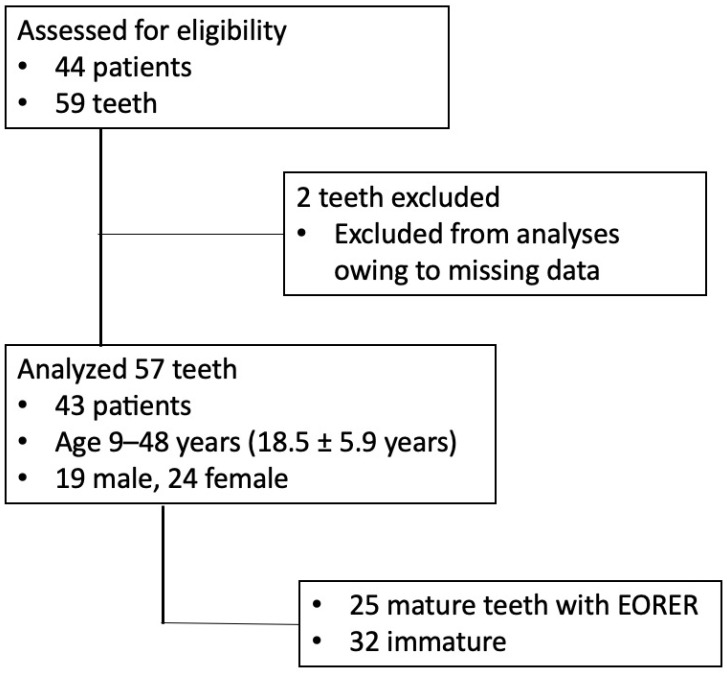
Strobe diagram representing the study patients and teeth.

**Figure 2:**
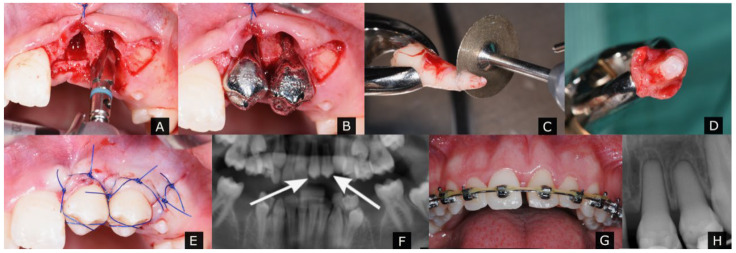
Autotransplantation of two mature premolars to the anterior left maxilla. (**A**) Recipient site preparation. (**B**) Tooth replica. (**C**) Root-end resection. (**D**) Enlarged diameter of the apical foramen. (**E**) Postoperative clinical image. (**F**) Postoperative radiograph image with arrows ranging from the donor to the recipient regions. (**G**) 4-year follow-up. (**H**) Intraoral radiograph 4 years after surgery with pulp obliteration.

During the study period, 59 teeth in 44 patients were transplanted. Two teeth were excluded owing to missing data. Consequently, 57 teeth in 43 patients were analyzed. The observation period was 12–68 months, with a mean follow-up of 21.2 ± 16.1 months. Overall, 32 teeth were immature (IT) and 25 teeth had completed root development (mature teeth, MT) (Figure 1). EORER was performed in the 25 MT (Figure 2).

### 3.1. Primary Outcomes

At the time of data evaluation, 55/57 examined teeth remained in situ (96.5%, MT 24/25, IT 31/32). PRV failed in 3/57 teeth (5.3%; IT 1, MT 2), which did not show positive perfusion MRI findings, radiologically detectable PCO, or clinically restored sensitivity.

PCO occurred in 46/57 teeth (80.7%), and PCO occurred significantly more in IT than in MT (IT 31/32 vs. MT 15/25; *p* = 0.001, Fisher’s exact test). In teeth with no PCO, MRI indicated PRV in five cases. Restored sensitivity was observed and clinical and/or radiological signs of pulpal necrosis were absent in three cases. PCO and PRV were absent in two mature maxillary second premolars transplanted to the mandibular premolar region and one immature maxillary premolar transplanted in the maxillary front. In these patients, signs of inflammatory root resorption were observed and endodontic treatment was initiated. In the latter case, the tooth was extracted after 4 years. Nine teeth (15.8%) exhibited signs of root resorption (MT 6/25, IT 3/32). Missing PRV was significantly associated with root resorption (*p* = 0.003, Fisher’s exact test). (Table 1)

**Figure 3:**
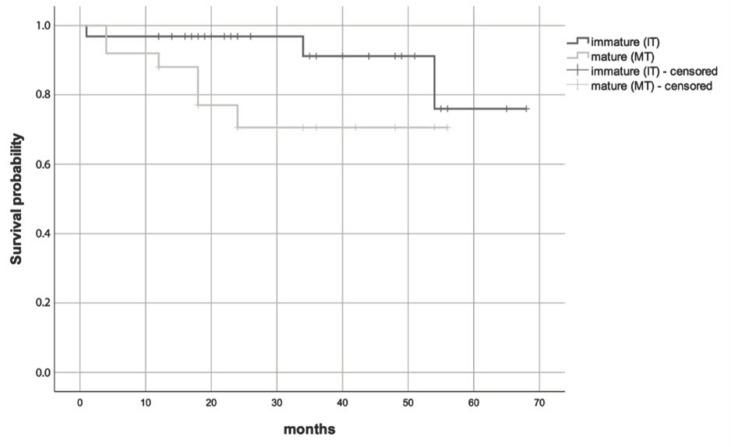
Comparison between the autotransplantation of immature (IT) and mature teeth (MT)—resorption-free survival.

The probabilities of resorption-free survival were 70.6% after 56 months in MT and 76% after 68 months in IT. No significant differences in resorption-free survival were detected between MT and IT (*p* = 0.077, logrank test); however, MT tended to resorb earlier (Figure 3).

### 3.2. Secondary Outcomes

All patients underwent preoperative clinical and radiological analyses. Radiological planning included the fabrication of a 3D replica of the tooth transplant based on segmented CBCT data. During surgery, the 3D replica was used to validate sufficient recipient site preparation. In six cases, the transplant was stored in a nutrition media (DENTOSAFE^®^, Medice, Iserlohn, Germany) during the preparation. Extra-oral storage was not associated with root resorption (*p* = 0.237, Fisher’s exact test, Table 1).

Interventions lasted 24–73 min (mean, 43 ± 12.7 min). Longer surgical durations were significantly associated with a higher incidence of root resorption (*p* = 0.01, *t*-test for independent samples) (Table 2). The EORER of MT did not lead to significantly longer surgeries (*p* = 0.05; *t*-test for independent samples).

The postoperative splinting of the transplant was maintained for 4–12 weeks. The duration of splinting did not influence the probability of root resorption (*p* = 0.607, *t*-test for independent samples) (Table 2).

The age of the patients at the time of surgery ranged from 9 to 48 years (mean, 17.6 ± 8 years) (Table 2). The physical status of all the patients was good (ASA 1–2), and only two patients were smokers. Patient age, sex, and health and smoking statuses did not significantly influence root resorption or PRV.

Most transplanted teeth were premolars (30/57, 52.6%), followed by molars (20/57, 35.1%). In the MT group, donors were most frequently molars (11/25, 44%) followed by premolars (10/25, 40%). Twenty-six grafts were fully or partially impacted. Tooth type, multiple roots, and impaction did not significantly affect outcomes (Chi-square and Fisher’s exact tests). (Table 1, Table 2 and Table 3) The apical diameter ranged from 0.6 to 4.4 mm (mean, 2.2 ± 1.1 mm) and did not significantly influence PRV or root resorption. (Table 2).

Sensitivity, which was confirmed using electric pulp or carbon dioxide snow, recovered in 92.9% teeth (53/57 teeth; MT 22/25, IT 31/32) from 4 to 40 weeks after surgery. Nevertheless, one sensitive mature tooth was lost 18 months after transplantation owing to external cervical root resorption. Sensitivity was not recovered in four cases, including three teeth (MT 2, IT 1) that were not revascularized. Sensitivity correlated significantly with revascularization (*p* < 0.001, Fisher’s exact test).

Following apical resection, the resulting root length in MT was two-third to three-fourth of the original length (23/25 teeth, 92%). Two teeth underwent a larger resection, resulting in approximately half the original root length. IT grew following transplantation (16/32 teeth, 50%), remained the same (12/32, 37.5%), or lost length (4/32, 12.5%). MT lost 3.26 mm (± 1.4 mm) and IT gained 1.9 mm (± 1.4 mm) of the root length, resulting in a similar root length of two-thirds to three-fourths of the expected original length.

## 4. Discussion

Our results showed that survival, root resorption, and pulp revascularization were similar in the AT of IT and MT (with EORER). Although the AT of IT is a routine procedure, that of IT with EORER has rarely been reported; however, the procedure has been described in animal studies since the 1980s [19,20]. Recently, a few studies reported that the AT of MT with EORER constitutes a feasible treatment option. To the best of our knowledge, this is the first study to compare root resorption and pulp revascularization in nearly similar-sized groups of mature and immature transplanted teeth.

The revascularization rates in IT with open apices are consistently high [21,22,23], which can be due to several factors. The wide diameter of the apical opening may facilitate cell migration and ingrowth of new vessels into the ischemic pulp space of a short-rooted tooth with simple root anatomy. In addition, stem cells in the apical papilla may drive the revascularization process. According to Andreasen et al. [21], the required diameter of the apical foramen to ensure successful revascularization is 1 mm, but apical sizes of <1 mm and even an apical foramen of 0.32 mm did not prevent tissue ingrowth in dogs [24]. A 2018 review by Fang et al. [12] included 14 studies and calculated the highest success rates for teeth with apical diameters of 0.5–1.0 mm; however, their results were not significant.

The anatomical changes of the root due to EORER, including a larger apical foramen, shorter root, and simplified root canal configuration, may contribute to the relatively high rate of PRV (92%, 23/25; Table 3) in mature transplanted teeth. Herein, a wide apical opening was not associated with PRV. However, the minimum diameter of MT was 0.6 mm. All teeth exceeded the threshold of 0.5 mm specified by Fang et al. [12] owing to surgical resection of the apical constriction in MT.

In a recent retrospective study by Raabe et al. [18] included nine major transplanted and root-end resected teeth, with four remaining without postoperative findings (resorption, failure, and apico-marginal lesion) after 1.3–5.3 years. A 5-year survival probability of 50% (95% CI: 12.5–100%) was calculated. Most failures occurred in teeth with more than one root canal. Likewise, in a meta-analysis by Chung et al. [25], posterior donors were associated with a higher risk of failure (3.3%) compared with premolars (1.6%) and anterior donor teeth (0.6%). Thus, PRV is more likely to occur in teeth exhibiting simple root morphology. Furthermore, a multirooted tooth might evoke greater extraction and transplantation trauma, and consequently, be more susceptible to periodontal complications. According to Aoyama et al. [26], multirooted donors were associated with significantly higher failure rates (Hazard ratio 1–52.5 for single-rooted versus 3.2–170.1 for multirooted teeth). However, this was not supported by the findings of the present study. More complicated root anatomy might be associated with longer surgical durations, a factor that was significantly related to higher root resorption rates. However, this assumption was also not supported by the current study. The presence of multiple roots or canals did not influence the main outcome variables.

Prolonged and more difficult surgery can contribute to additional stress to the periodontal ligament and, thus, threaten the viability of the periodontal ligament cells [9]. In this context, the experience of surgeons might also be essential as the careful handling of the donor tooth is extremely important to minimize damage [8,27]. All four oral surgeons who performed the procedures in the current study were highly experienced.

Extra-oral time of the transplant can significantly influence the success rate of AT [28]. Consequently, applications for creating tooth replicas with high accuracy have been developed [16,29,30]. They can be used a model to validate the correct preparation of the recipient bed. Thus, the transplant can be reinserted immediately and as gently as possible after the extraction. If the preparation of the recipient site can only be performed after donor tooth removal, extra-oral storage is inevitable. Storing donor tooth in a cell nutrition media might diminish the negative effect of extraoral time on periodontal healing as it does not increase the risk of root resorption.

Constant liquid cooling was applied to diminish heat-induced trauma via root tip resection. This might damage periodontium focal cells and cause focal ankylosis in the further course. However, this was not observed in the current study. The provocation of thermal damage to the pulp cells might be less relevant as pulp necrosis occurs because of blood supply disruption. It has been shown that the odontoblastic layer rarely survives after transplantation or regenerative endodontic treatment [15,31].

Several clinical and/or radiological signs indicate PRV. Radiologically, PRV can be visualized directly via perfusion MRI and indirectly via PCO. Perfusion MRI is an invasive and strenuous procedure and is thus considered as an overtreatment following the AT of IT in children where PRV is more likely to occur. Conversely, in uncertain cases, it is a useful tool as it can bring immediate clarity. This applies to MT transplantation. However, MRI does not provide interpretable results if artifact-causing elements, such as orthodontic devices, are present. Allergy to the contrast agent and claustrophobia are further contraindications. Therefore, it was only performed if reasonable. The revascularization rate was 94.7% (54/57 teeth) in our study; however, PCO was only present in 80.7% teeth (46/57 teeth). The lower PCO rate was most likely due to different types of wound repair and the resulting intracanal tissue. In the cases of the AT of MT with EORER and no apical papilla, PCO might also be caused by the ingrowth of cementum and bone, as observed by Yamauchi et al. [31], or even just fibrovascular tissue [32] instead of reparative dentin. Herein, PCO was significantly less common in MT than in IT.

Age, sex, and general health did not influence the outcome of this study. This might be attributed to proper case selection as only healthy individuals were suitable candidates for AT.

The short follow-up time is a major shortcoming of the present study, as the endodontic success rate may drop with longer observation periods. Murtadha and Kwok [33] concluded in their retrospective analysis, which included 252 teeth, that external root resorption and periapical pathology, which are the major reasons for endodontic therapy, occurred predominantly within the first 3 years. Thus, intensified aftercare for 3 years was recommended. Nevertheless, the high revascularization rate in MT after at least 1 year in this cohort was remarkable.

Further shortcomings are due to the retrospective design of the study. Even though patients were recruited consecutively, recall or misclassification bias might influence the composition of the groups. Furthermore, possible confounding factors might not have been measured or documented.

Another limitation of this study is the rather small cohort. In the meta-analysis conducted by Almpani and Kolokitha [34], smaller studies with <100 teeth tended to overestimate the event rates. A larger study cohort might produce more distinctive results regarding possible confounders. Nevertheless, early results concerning an evolving technique are important to estimate its feasibility. Furthermore, this study may serve as a foundation for well-designed larger trials.

## 5. Conclusions

We demonstrated that the resection of the apical constriction in mature transplanted teeth yielded similar results in terms of revascularization and root resorption as in those of immature transplanted teeth as long the cases were selected appropriately.

Therefore, the transplantation of MT might be a feasible treatment option. The results of the present study also indicate that EORER is a potential technique for promoting pulpal healing in mature transplanted teeth. Longer surgical durations and failing revascularization increased the rates of root resorption, thereby impairing the prognosis. Further factors should be evaluated in larger trials with longer observation periods to confirm these results. Basic research examining tissue regeneration after the AT of MT with EORER remains lacking.

## Figures and Tables

**Table 1:** Root resorption, part 1.

*Root Resorption n/n*	*MT n/n*	*IT n/n*	p-Value *
9/57	6/25	3/32	*p* = 0.128
	*Impacted*	*Not impacted*	
	4/26	5/31	*p* = 0.615
	*Extraoral storage*	*No extraoral storage*	
	2/6	7/51	*p* = 0.237
	*Multirooted*	*Single-rooted*	
	3/15	6/42	*p* = 0.439
	*PRV*	*No PRV*	
	6/54	3/3	***p* = 0.003**

n/n = number of cases from total cases, MT = mature teeth, IT = immature teeth, significance levels (* Fisher’s Exact test), bold = statistically significant.

**Table 2:** Root resorption, part 2.

*Root Resorption*	*Yes n = 9*	*No n = 48*	p-Value *	
*Age*	19.89 ± 8.04	17.21 ±8.04	*p* = 0.378	*t-test for independent samples*
*Duration of surgery (min)*	52.89 ± 10.74	41.19 ± 12.24	***p* = 0.010**	
*Splinting (weeks)*	5.50 ± 2.98	5.11 ± 1.77	*p* = 0.607	
*Apical diameter (mm)*	2.14 ± 1.02	2.30 ± 1.25	*p* = 0.728	
***Donor type***				
*Canine*	2/4	2/4	*p* = 0.297	*Chi-square test*
*Max. molar*	2/12	10/12		
*Max. premolar*	4/26	22/26		
*Mand. molar*	1/8	7/8		
*Mand. premolar*	0/7	7/7		

n = number of cases, Max. = maxillary, Mand. = mandibular, significance levels (* *t*-test for independent samples, Chi-square test), bold = statistically significant.

**Table 3:** Pulpal revascularization.

*PRV n/n*	*MT n/n*	*IT n/n*	p-Value *
54/57	23/25	31/32	*p* = 0.407
	Impacted	Not impacted	
	26/26	28/31	*p* = 0.154
	Extra-oral storage	No extra-oral storage	
	6/6	48/51	*p* = 0.712
	Multi-rooted	Single-rooted	
	15/15	39/42	*p* = 0.392

n/n = number of cases from total cases, MT = mature teeth, IT = immature teeth, significance levels (* Fisher’s Exact test).

## Data Availability

The data that support the findings of this study are available from the corresponding author upon reasonable request. The data are not publicly available due to privacy or ethical restrictions.
